# Expressions of miR‐525‐3p and its target gene *SEMG1* in the spermatozoa of patients with asthenozoospermia

**DOI:** 10.1111/andr.12573

**Published:** 2018-12-21

**Authors:** Q.‐z. Zhou, X.‐b. Guo, W.‐s. Zhang, J.‐h. Zhou, C. Yang, J. Bian, M.‐k. Chen, W.‐b. Guo, P. Wang, T. Qi, C.‐y. Wang, J.‐k. Yang, C.‐d. Liu

**Affiliations:** ^1^ Department of Urology The Third Affiliated Hospital of Southern Medical University Guangzhou China; ^2^ Department of Laboratory Medicine Nanfang Hospital Southern Medical University Guangzhou China

**Keywords:** asthenozoospermia, male infertility, miR‐525‐3p, *SEMG1*

## Abstract

**Background:**

Semenogelin 1 (*SEMG1*) is an important secretory protein in spermatozoa involved in the formation of a gel matrix encasing ejaculated spermatozoa. Previous studies show that the SEMG1 gene is highly expressed in spermatozoa from patients with asthenozoospermia (AZS); however, the underlying molecular mechanisms are not yet clear.

**Objectives:**

To study the molecular mechanism of high expression of SEMG1 gene and its potential roles in AZS.

**Materials and Methods:**

Western blot and real‐time PCR were used to detect the expression levels of *SEMG1* protein and mRNA in the ejaculated spermatozoa from normozoospermic males and AZS patients. Bioinformatics analysis was used to predict miRNAs targeting for SEMG1 3′‐untranslated region detection of the expression levels of all the candidate miRNAs in ejaculatory spermatozoa in AZS patients or normozoospermic volunteers. Luciferase reporter assays were performed to confirm it can directly bind to SEMG1. Correlation of miR‐525‐3p and *SEMG1* mRNA expression with clinical sperm parameters were also analyzed. Finally, we conducted a follow‐up study of reproductive history about all the subjects.

**Results:**

SEMG1 mRNA and protein level were significantly higher in AZS patients compared to that in normozoospermic volunteers (*p *<* *0.001). Subsequently, microRNA‐525‐3p (miR‐525‐3p) which was predicted as a candidate regulator of *SEMG1* was found lower expressed in ejaculatory spermatozoa in AZS patients (*p* = 0.0074). Luciferase experiment revealed that microRNA‐525‐3p could directly target *SEMG1* 3′‐untranslated region and suppress its expression. Importantly, our retrospective follow‐up study showed that both low miR‐525‐3p expression and high SEMG1 expression level was significantly associated with low progressive sperm motility, abnormal sperm morphology, and infertility.

**Discussion and Conclusion:**

The elevated expression of SEMG1 and reduced expression of miR‐525‐3p are associated with AZS and male infertility. Our study provides a potential therapeutic target for the treatment of male infertility or for male contraception.

## Introduction

Infertility has become a global reproductive health problem, affecting approximately 10%‐15% of couples of childbearing age, with the men responsible for approximately half of the cases (Gilany *et al*., 2014; Esteves, 2016). Awfully, the sperm density and sperm activity decrease yearly at a certain rate, which may be associated with the increasing environmental pollution (Santi *et al*., 2018). Asthenozoospermia (AZS), one of the common causes of male infertility, is characterized by the reduction or absent of sperm motility, with normal sperm morphology in fresh ejaculate according to the World Health Organization (WHO) guidelines (5 th ed.). Several reasons may account for AZS, such as urogenital infections, abnormal varicocoele, semen liquefaction, abnormal hormones, and so on (Abbasihormozi *et al*., 2017; Giacone *et al*., 2017). However, approximately 50% of male infertility cases cannot find an exact etiological factor, which are referred to as idiopathic male infertility (Gilany *et al*., 2014). The pathogenesis of idiopathic AZS remains largely unknown, which hinders the progress of diagnosis and treatment of AZS.

Recently, several studies have demonstrated that some differentially expressed genes involved in the molecular mechanism of AZS such as cilia‐and flagella‐associated protein 69 (CFAP69) and cysteine‐rich secretory protein 2 (*CRISP2*), have been reported to be associated with AZS in mice or human (Zhou *et al*., 2017; Dong *et al*., 2018). Furthermore, our previous study has identified SEMG1 as a potential marker for idiopathic asthenozoospermia (Yu *et al*., 2014). SEMG1, the major secretory protein in male semen, originates from seminal vesicles and is involved in the formation of gel matrix that encases ejaculated spermatozoa. After ejaculation, SEMG1 was degraded into smaller peptides by prostate‐specific antigen (PSA), which allows the spermatozoa to get motile ability and move quickly (Robert & Gagnon, 1996). Martínez‐Heredia *et al*. found SEMG1 protein was highly expressed in AZS semen samples using two‐dimensional proteomic analysis (Robert & Gagnon, 1999; Martinez‐Heredia *et al*., 2008). In addition, our previous study found an over‐expression of *SEMG1* in AZS patients through gene expression profile analyses of AZS patients and volunteers spermatozoa, indicating an important role of *SEMG1* in AZS (Yu *et al*., 2014). However, the molecular mechanism underlying over‐expression of *SEMG1* in patients with AZS remains unclear.

In this study, we analyzed the expression of SEMG1 and miR‐525‐3p in the ejaculated spermatozoa from patients with AZS and normozoospermic volunteers. Furthermore, we identified whether miR‐525‐3p has the regulatory effects on the expression of SEMG1 in AZS. This may provide the opportunity to further illustrate the molecular mechanisms of AZS. Meanwhile, a follow‐up study of reproductive history indicated that both low expression of miR‐525‐3p and high expression of SEMG1 were correlated with low progressive sperm motility, abnormal morphology, and infertility, respectively. Our findings demonstrate that the deletion of miR‐525‐3p contributes to the aberrant expression of *SEMG1*, clinically involving in AZS and male infertility, which provides a potential therapeutic target for the treatment of male infertility and for male contraception.

## Materials and Methods

### Ethics statement

Our study was approved by the Bioethics Committee of Nanfang Hospital and the Third Affiliated Hospital of Southern Medical University. Informed consent was obtained from all subjects.

### Collection and preparation of human sperm samples

A total of 30 patients with AZS in Nanfang Hospital from January 2017 to May 2017 were enrolled in the present study. During the same period, 30 age‐matched normozoospermic volunteers in the same Hospital were enrolled. According to the World Health Organization (WHO) guidelines (5th ed.), AZS is defined as having <32% progressive motility rate in fresh semen samples and confirmed by routine semen analysis of three times semen samples collected at different time points. After 3 to 7 days of abstinence, fresh semen samples were obtained from all study subjects by masturbation and then liquefied at 37 °C. for 30 min. Participants with varicocoele, teratozoospermia, leukocytospermia, reproduction tract infections, abnormal hormones (such as testosterone, follicle‐stimulating hormone, estradiol), and a history of cryptorchidism, orchitis, or epididymitis were excluded. In addition, samples with abnormalities of semen parameters such as semen liquefaction, pH, α‐glucosidase, acid phosphatase, seminal fructose, and anti‐sperm antibody (+) were also excluded.

All liquefied semen samples were analyzed for the primary semen parameters using Sperm Class Analyzer (SCA, Microptic, Barcelona, Spain) and evaluated for sperm viability by Eosin staining. For the estimation of sperm morphology, semen samples were stained with Diff‐Quick (Dade Behring, Newark, NJ, USA). Liquefied semen samples mixed with 50% Percoll density gradient centrifugation (Pharmacia, Uppsala, Sweden) and then centrifuged at 2000 g for 15 min at room temperature (Mengual *et al*., 2003; Zhou *et al*., 2010). Separated and purified spermatozoa were stored at −80 °C until further use.

### RNA extraction, reverse transcription, and quantitative real‐time PCR

Total RNA, including microRNAs (miRNAs), was isolated from sperm pellets using TRIzol^®^ reagent (Invitrogen, Carlsbad, CA, USA). The quality and concentration of extracted total RNA were measured spectrophotometrically at optical density (OD) 260/280 and 260/230 with NanoDrop ND‐1000 spectrophotometer (Thermo Scientific, Waltham, MA, USA). RNA was reverse transcribed by PrimerScript™ RT Kit (TaKaRa, Dalian, China) for mRNA and by SYBR^®^ PrimeScript™ miRNA RT‐PCR Kit (TaKaRa) for miRNA. Extraction and reverse transcription of RNA were performed according to the manufacturer's instruction.

Quantitative real‐time PCR (qRT‐PCR) was performed using SYBR^®^ Premix Ex Taq™ RT‐PCR Kit (TaKaRa) for mRNA in Mx3005P qRT‐PCR System (Stratagene, Santa Clara, CA, USA). Amplification reactions were performed according to manufacturer's instructions. qRT‐PCR was performed in triplicate, and human glyceraldehyde‐3‐phosphate dehydrogenase(*GAPDH*) was served as an endogenous control.

The qRT‐PCR was measured using SYBR^®^ PrimeScript™ miRNA RT‐PCR Kit for miRNA in Mx3005P qRT‐PCR System. Amplification reactions were performed according to manufacturer's instructions. qRT‐PCR was performed in triplicate, and levels of miRNAs were normalized relative to the levels of RNU6B small nuclear RNA as an endogenous control. Relative mRNA and miRNA expressions were calculated using the comparative Ct (2‐^ΔΔCt^) method. Primers are shown in Table 1.

### Prediction of miRNAs targeting *SEMG1*

We used miR Walk database (http://www.umm.uni-heidelberg.de/apps/zmf/mirwalk) to predict miRNAs that may bind to 3′ untranslated region (3′UTR) of SEMG1.

### Western blot analysis

Sperm and HEK293T cells were lysed on ice with 100 μL lysis buffer and 1 μL protease inhibitor (total protein extraction kit, BestBio, Shanghai, China) for 30 min, respectively. Subsequently, proteins were extracted according to the manufacturer's instructions. Thirty ug of total protein was electrophoresed on 12% (w/v) SDS‐PAGE gels (80V, 30 min; 120 V, 75 min) and transferred to PVDF membranes (BioTrace, Pall, Mexico) (320 mA, 120 min). Blots were blocked in 5% non‐fat dry milk dissolved in PBS and incubated with antibodies against SEMG1 (1:1000, ab139405, abcam) or GAPDH (1:10000, ab8245, abcam) at 4 °C overnight. Anti‐rabbit or anti‐mouse IgG HRP‐conjugated antibodies (1:10000, GreatOcean) were used for 30 min at room temperature. We used the Enhanced Chemiluminescence kit (Guangzhou, China) to display protein bands. The intensities of protein bands were quantified by image lab software and normalized to that of GAPDH.

### Dual luciferase activity assay

The 3′‐UTR of human *SEMG1* containing putative binding sites for miR‐525‐3p was cloned into a luciferase reporter vector. Wild‐type pMIR‐REPORT ‐ SEMG1 ‐ 3′UTR (H8361) and mutant luciferase reporter pMIR‐REPORT ‐ *SEMG1* ‐ 3′UTR (H8362) were generated by Obio Technology (Shanghai, China). HEK 293T cells were seeded in 96‐well plates and cultured to 70% confluence. HEK 293T cells were transfected with luciferase reporter vector and miR‐525‐3p or miRNA precursor as a negative control. Luciferase activity was detected 72 h after transfection using Dual Luciferase Reporter Assay kit (Promega) according to the manufacturer's instructions. Each experiment was repeated at least three times.

### Follow‐up study of reproductive history

To assess the effect of SEMG1 mRNA and miR‐525‐3p expression levels on fertility, we conducted a follow‐up study of reproductive history. Subjects were divided into relatively high and low expression groups based on the SEMG1 mRNA and miR‐525‐3p expression levels. Male infertility is defined as inability of a sexually active, non‐contraceptive couple to achieve pregnancy due to male factors in 1 year (Dohle *et al*., 2005).

### Statistical analysis

We applied spss software version 22.0 (Chicago, IL, USA) and graphpad prism software version 5.0 (San Diego, CA, USA) for statistical analysis. Data are presented as $\overset{¯}{x}$ *± s*, and *p *<* *0.05 was considered statistically significant. We used Independent Student's *t*‐test to compare the differences of *SEMG1* mRNA and miR‐525‐3p expression in the ejaculated spermatozoa between AZS and normozoospermic males. Significant differences in luciferase activity were also determined by using Student's *t*‐test. Spearman correlation was used to assess the associations between the expression level of *SEMG1* mRNA, miR‐525‐3p, and clinical features of semen samples. Chi‐square or Fisher's exact test was used to analyze the differences in fertility rates between different groups.

## Results

### Comparison of the basic parameters of semen in AZS patients and normozoospermic volunteers

We compared the semen routine parameters of 30 AZS patients and 30 age‐matched normal sperm volunteers. There was no significant difference between patients with AZS and normozoospermic volunteers in terms of age, sperm volume, pH, and sperm concentration. Notably, sperm progressive motility and normal morphology in AZS patients was lower than that in normozoospermia control group (*p *<* *0.001, Table 2).

### SEMG1 mRNA and protein expression in the ejaculated spermatozoa of AZS patients and normozoospermic volunteers

**Figure 1:**
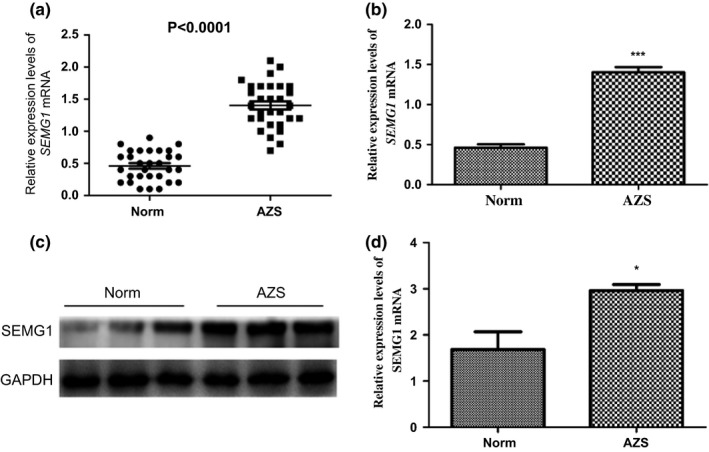
The mRNA and protein expression levels of *SEMG1* were examined by qRT‐PCR and Western blots, respectively. (a, b) The *SEMG1* mRNA levels were significantly higher in AZS patients compared with that in normozoospermic volunteers (1.40 ± 0.35 vs. 0.46 ± 0.23, *p *<* *0.001). (c, d) The protein expression of SEMG1 in the spermatozoa of AZS patients was significantly higher than that in normozoospermic controls (2.96 ± 0.13 vs. 1.68 ± 0.38, *p <* *0.005 and ***p <* *0.001).

The expression of SEMG1 mRNA and protein was detected and analyzed in the spermatozoa of AZS patients and normozoospermic volunteers. The qRT‐PCR results showed that the *SEMG1* mRNA level was significantly higher in AZS patients compared to that in normozoospermic volunteers (1.40 ± 0.35 vs. 0.46 ± 0.23, *p *<* *0.001, Fig. 1a,b). Similarly, the Western blot analysis of the *SEMG1* protein also showed that the expression of *SEMG1* protein was significantly higher in the spermatozoa of AZS patients than those of normozoospermic controls (2.96 ± 0.13 vs. 1.68 ± 0.38, *p *<* *0.001, Fig. 1c,d).

### miR‐525‐3p directly regulated *SEMG1* expression by binding to its 3′‐UTR and its expression was lower in the ejaculated spermatozoa of AZS

**Figure 2:**
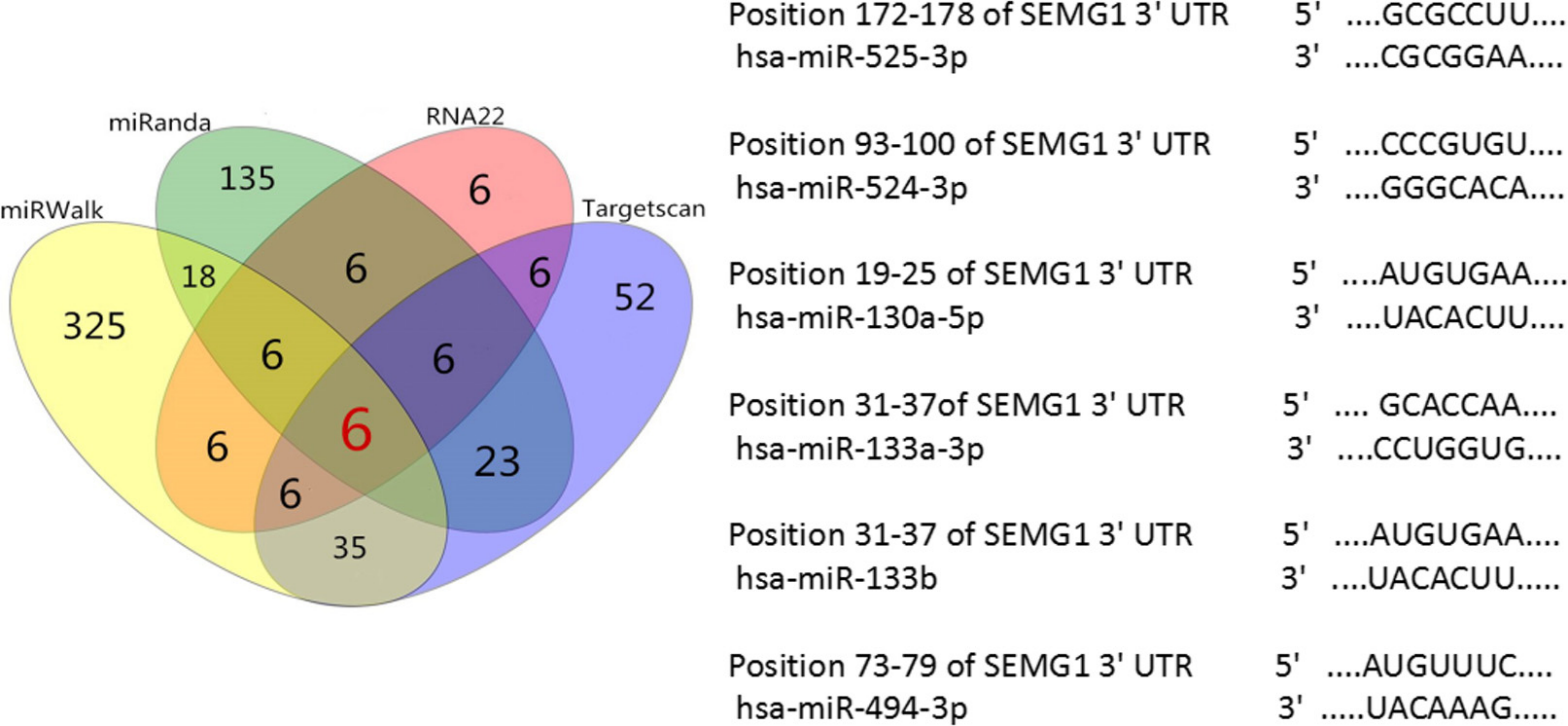
Six candidate microRNAs were predicted by miRWalk database to potentially bind to the *SEMG1* 3′UTR. [Colour figure can be viewed at wileyonlinelibrary.com]

**Figure 3:**
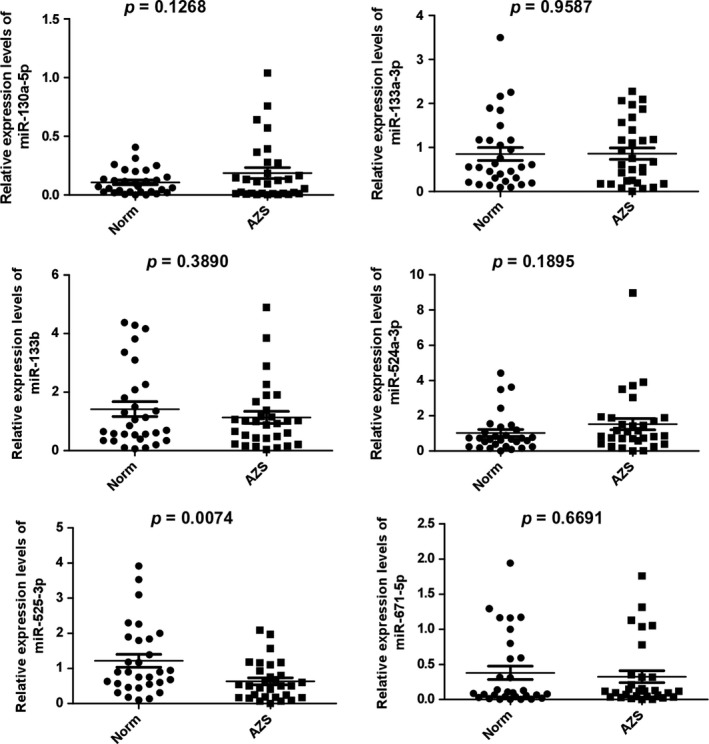
The miR‐525‐3p levels were significantly lower in AZS patients compared to that in normozoospermic volunteers (1.2 ± 1.00 vs. 0.64 ± 0.55, *p *=* *0.0074).

We used miRWalk database to predict miRNAs potentially regulating *SEMG1* gene. As a result, six candidate miRNAs (miR‐525‐3p, miR‐524‐3p, miR‐133b, miR‐671‐5p, miR‐133a‐3p, and miR‐130a‐5p) were selected (Fig. 2). qRT‐PCR was used to validate the expression level of selected miRNAs. Interestingly, among of these miRNAs, only miR‐525‐3p was lowly expressed in the ejaculated spermatozoa of AZS patients compared with that in normozoospermic controls (1.22 ± 1.00 vs. 0.64 ± 0.55, *p *=* *0.007, Fig. 3), implying a potential association between miR‐525‐3p and *SEMG1* in AZS.

**Figure 4:**
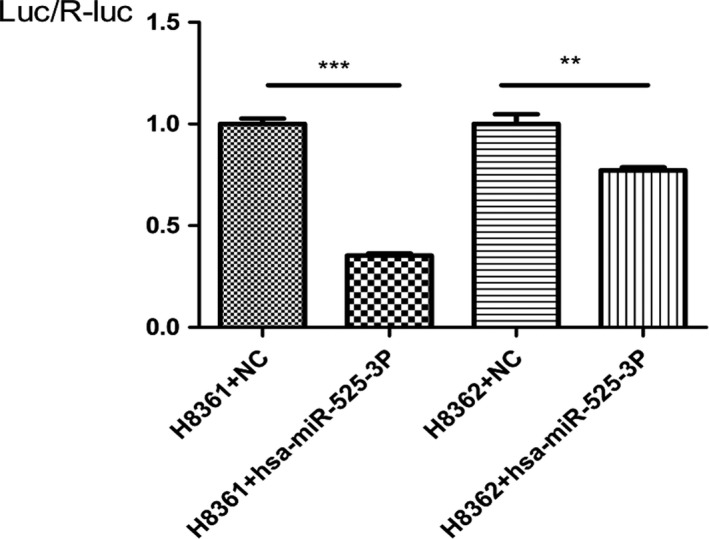
Results of luciferase reporter assay in HEK 293T cells with co‐transfection of 3′‐UTR vector or NC vector. The bar graph shows the mean ± s.d. in the three independent experiments. One‐way ANOVA and Bonferroni test are carried out to determine significant differences in luciferase activity (***p <* *0.001 and **p <* *0.01). SEMG1: semenogelin 1; NC: negative control; s.d.: standard deviation.; H8361: Wild‐type pMIR‐REPORT ‐ SEMG1 ‐ 3′UTR; H8362: mutant pMIR‐REPORT ‐ SEMG1 ‐ 3′UTR.

To further confirm whether miR‐525‐3p could specifically regulate *SEMG1* expression, we performed luciferase activity assays. When co‐transfected 293T cells with miR‐525‐3p mimic and plasmids, a clear suppression of luciferase activity was observed for both the wild‐type and the mutant construct (Fig. 4). Our results confirmed that miR‐525‐3p could directly regulate *SEMG1* by binding to its 3′‐UTR.

### Correlation of miR‐525‐3p and *SEMG1* mRNA expression with clinical sperm parameters

**Figure 5:**
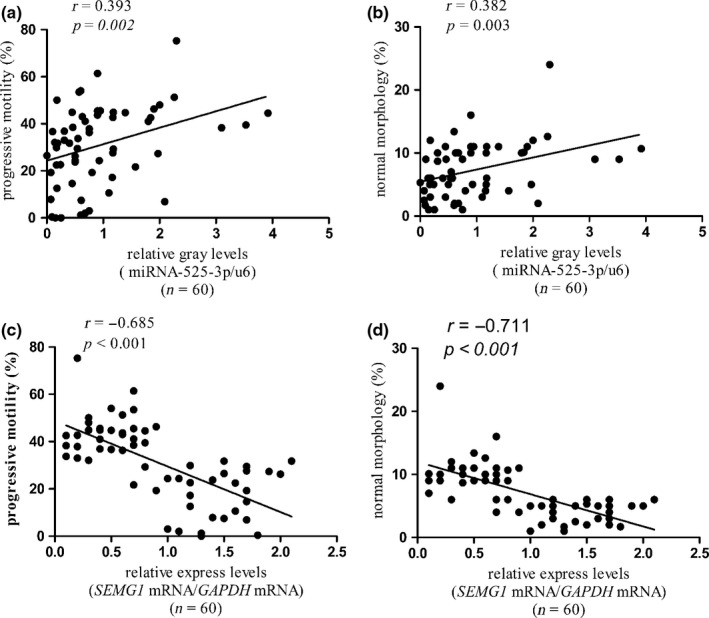
Relatively low miR‐525‐3p expression or high *SEMG1* mRNA expression is correlated with reduced sperm motility and abnormal sperm morphology. (a, b) The expression of miR‐525‐3p expression was positively correlated with sperm progressive motility and normal morphology. (c, d) The expression level of *SEMG1* mRNA was negatively correlated with sperm progressive motility and normal morphology.

We analyzed the correlation between the expression of miR‐525‐3p or *SEMG1* mRNA and clinical sperm parameters. The results illustrated that miR‐525‐3p expression was positively correlated with sperm progressive motility (*r* = 0.393, *p *=* *0.002, Fig. 5a), normal morphology (*r* = 0.382, *p *=* *0.003, Fig. 5b). On the contrary, the expression level of *SEMG1* mRNA was negatively correlated with sperm progressive motility (*r* = −0.685, *p *<* *0.001, Fig. 5c) and normal morphology (*r* = −0.711, *p *<* *0.001, Fig. 5d). Furthermore, the relative expression of miR‐525‐3p or *SEMG1* mRNA has no correlation with other clinical sperm parameters, such as age, semen volume, and sperm concentration (*p *>* *0.05).

### Correlation of miR‐525‐3p and *SEMG1* mRNA expression with fertility

**Figure 6:**
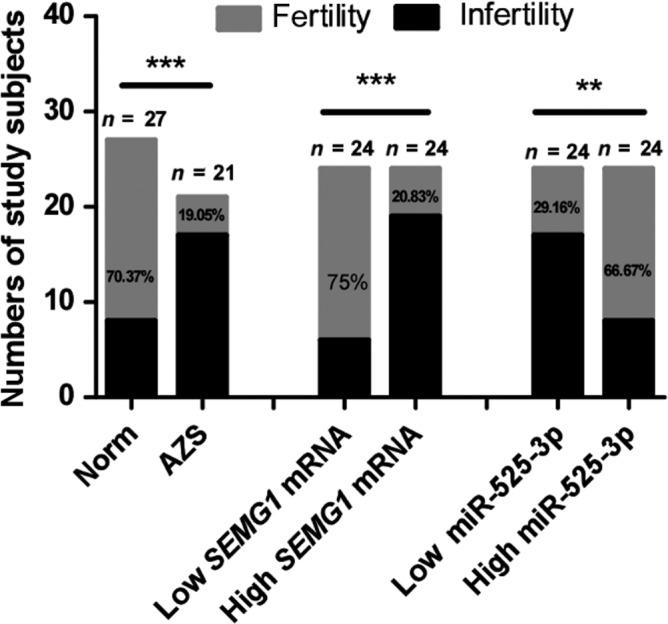
The fertility rates were displayed in the indicated groups. Fisher's exact test was used to assess the statistical significance of infertility rate differences between different groups (*p <* *0.05,**p <* *0.01). SEMG1: semenogelin 1; Norm: normozoospermic control group; AZS: asthenozoospermic patients group.

To explore the clinical relevance of miR‐525‐3p or *SEMG1* mRNA expression to AZS, we conducted a retrospective follow‐up study of the reproductive history of all subjects. Of these subjects, 12 subjects were excluded from our study. Excluded subjects included lost contact or refused to cooperate (*n* = 9), unmarried (*n* = 1), and female partner infertility (*n* = 2). Overall, 21 AZS patients and 27 normozoospermic volunteers were included in this study (Table 3). Subsequently, we grouped the included subjects into relatively high and low expression groups according to the expression levels of miR‐525‐3p or *SEMG1* mRNA in their spermatozoa. As shown in Fig. 6, the fertility rate was higher in normozoospermic volunteers than those in AZS patient (70.37% vs. 19.05%, *p *<* *0.001). Of note, a higher fertility rate was observed in relatively low *SEMG1* mRNA expression group (75% vs. 20.83% *p *<* *0.001) and relatively high miR‐525‐3p expression group (66.67% vs. 29.16% *p *<* *0.001).

## Discussion

SEMG1 is a major protein of semen coagulum that has been shown to inhibit human sperm capacitation (de Lamirande *et al*., 2001a,2001b). Clinical evidence showed that SEMG1 plays roles in modulating semen liquefaction, sperm capacitation, and regulation of sperm cell membrane permeability (Robert & Gagnon, 1999; de Lamirande *et al*., 2001a; Mitra *et al*., 2010). In this study, we found that the mRNA and protein of SEMG1 were highly expressed in the spermatozoa of AZS patients. SEMG1 is mainly expressed in the prostate and little express in germ cells(Robert & Gagnon, 1999; de Lamirande *et al*., 2001a,2001b). How is SEMG1 mRNA incorporated in ejaculated spermatozoa? The maturation of sperm cells is a long multi‐step biological process as the male gamete, when released from the seminiferous epithelium in the testis, they are not able to naturally fertilize an oocyte. This process demands modification of their metabolism, membrane and intracellular structures, and biochemical composition. One of the key actors in post‐testicular sperm maturation is represented by several types of lipid vesicles secreted by the epithelium of the male reproductive tract glands, interacting sequentially with the gametes, namely the epididymosomes and the prostasomes. These vesicles allow the acquisition of a particular protein, RNA and a lipid composition that are fundamental for the steps of gamete recognition and fusion (Sullivan & Saez, 2013). The prostatsomes can transfer the substance to the spermatozoa, such as DNA and CD38(Park *et al*., 2011; Ronquist *et al*., 2011). Our previous study found that SEMG1 protein is contained in exosomes extracted from seminal plasma (Yang, *et al*., 2017). So we speculated that SEMG1 mRNA and protein could be incorporated in ejaculated spermatozoa by exosomes from prostate, though, further research is required to investigate it.

There have been extensive studies reported the miRNA expression profiles of testis in humans or animals, suggesting the important roles of miRNAs in the process of testis development and spermatogenesis (Yang *et al*., 2013; Wu *et al*., 2014). Recently research on spermatozoal miRNAs has made considerable progress. Ma J found that testosterone‐dependent miR‐26a‐5p and let‐7 g‐5p act as signaling mediators to regulate sperm apoptosis via targeting PTEN and PMAIP1(Ma *et al*., 2018). Furthermore, miRNAs control mRNA fate by compartmentalization based on 3′ UTR length in male germ cells (Zhang *et al*., 2017). In addition, Brohi RD showed that post‐translational modifications in spermatozoa have an effect on male fertility and sperm viability (Brohi & Huo, 2017). Recent research found that the expression of miR‐15a was significantly decreased in the ejaculated spermatozoa of patients with varicocoele and miR‐15a repressed the expression of *HSPA1B* through directly binding its 3′UTR (Ji *et al*., 2014). Consistently, our previous researches also observed that miR‐27b and miR‐27a could target the 3′UTR of *CRISP2* and down‐regulate its expression in AZS or asthenoteratozoospermia, respectively (Zhou *et al*., 2015, 2017). In this study, bioinformatics predicted that miR‐525‐3p could be a specific regulator of *SEMG1*. Dual luciferase activity assay further confirmed that miR‐525‐3p could directly bind to the 3′UTR of *SEMG1* and suppress its expression. Thus, we proposed that one of the mechanisms underlying *SEMG1* over‐expression in AZS is the deletion of miR‐525‐3p, though there may be other regulators contribute to *SEMG1* over‐expression, which deserves further investigation.

Study shows that miR‐525‐3p acts as an oncogenic miRNA in liver tumor, which enhances the migration and invasion of liver cancer cells (Pang *et al*., 2014). The expression level of miR‐525‐3p also increases in cisplatin‐resistant germ cell tumor cell lines (Port *et al*., 2011), suggesting miR‐525‐3p may positively relate to the malignancy of germ cell tumor cells. Nevertheless, there are no reports on the function and mechanism of miR‐525‐3p in AZS. It will be interesting to establish the extent by which this miRNA is expressed in the prostate or in another tissue. As mentioned in the literature, reduced expression of miR‐525‐3p was observed in semen of infertile men compared with normal semen (Liu *et al*., 2012). We all know that normal semen is a viscous liquid mixture consisting of sperm and seminal plasma, which accounts for more than 90% of the semen volume. The seminal plasma is a mixture mainly consisting of secretory glands secreted by the prostate, seminal vesicles and urethral glands, and also includes a small part of testicular fluid and epididymal fluid. In addition to a large amount of water, fructose, protein and peptides, seminal plasma contains a variety of other sugars (such as glucose), enzymes (such as prostaglandins), and small molecules, which can provide nutrition and energy for spermatozoa. Therefore, miRNA‐525‐3p may be derived from any part of the reproductive tract such as testis, epididymis, seminal vesicle, and prostate. We will further study the distribution of this miRNA in various tissues of the reproductive system and its role in male infertility. Relatively high expression of *SEMG1* mRNA or low expression of miR‐525‐3p in the spermatozoa was correlation with low progressive sperm motility and abnormal morphology, respectively, which is consistent with our current observations. SEMG1 is a major protein of semen coagulum that has been shown to inhibit human sperm capacitation. In our research, there were higher spermatozoa abnormal morphology rate with relatively high expression of mRNA *SEMG1* or low expression of miR‐525‐3p (Fig. 5b,d). More research is needed to confirm how SEMG1 influences sperm morphology. The relationship between high *SEMG1* mRNA or low miR‐525‐3p and male infertility was found in our follow‐up study of reproductive history. It is interesting to note that the infertility rate in AZS patients was higher while high infertility rate was closely related to high *SEMG1* or low miR‐525‐3p expression. Though there may be more unidentified regulatory mechanisms that may contribute to AZS and sterility, our research first demonstrates that miR‐525‐3p deletion may contribute to aberrant expression of *SEMG1*, clinically involving in AZS and male infertility. In this study, we analyzed the expression levels of miR‐525 and SEMG1 in human spermatozoa and found that they are closely related to AZS; however, larger sample studies and further experiments in vivo are needed to confirm our conclusions.

## Conclusion

Our study found that the expression of miR‐525‐3p in ejaculatory spermatozoa of AZS patients was lower, while SEMG1 expression was higher compared with normal spermatozoa. Furthermore, miR‐525‐3p deletion contributes to aberrant expression of SEMG1. Further clinical correlation analysis and follow‐up studies of the reproductive history of study patients suggested that elevated SEMG1 and reduced miR‐525‐3p levels are associated with AZS and male infertility. Our study provides a valuable insight into AZS and male infertility, which may help to provide a potential diagnostic marker and therapeutic target for treating male infertility or for male contraception.

## Conflict of Interest

There were no competing interests among the authors.

## Authors’ Contributions

CDL and JKY designed the study. TQ and CYW collected the semen samples. WSZ and PW performed the Western blot. MKC analyzed the experimental data. XBG and QZZ drafted the manuscript. WBG and JHZ participated in the revising of the manuscript. All authors read and approved the final manuscript.
